# A novel inflammasome-related gene nomogram predicts survival in hepatocellular carcinoma

**DOI:** 10.1097/MD.0000000000033121

**Published:** 2023-02-22

**Authors:** Zhengqi Lv, Heng Li, Yiwen Yuan, Qinghua Wu

**Affiliations:** a Wuxi School of Medicine, Jiangnan University, Wuxi, Jiangsu, P.R. China; b Guizhou Medical University, Guiyang, Guizhou, P.R. China; c Department of Radiology, Affiliated Hospital of Jiangnan University, Wuxi, Jiangsu, P.R. China.

**Keywords:** hepatocellular carcinoma, inflammasomes, prognosis prediction, tumor immune environment

## Abstract

Inflammasomes are closely associated with the progression of multiple cancers. We established an inflammasome-related gene (IRG)-based model to predict the survival of patients with hepatocellular carcinoma (HCC). The RNA-sequencing data and clinical information of HCC patients were downloaded from the cancer genome atlas-liver hepatocellular carcinoma database, and the differentially expressed inflammasome-related gene were screened. Seven prognostic differentially expressed inflammasome-related genes were identified by univariate Cox analysis and incorporated into the risk model using least absolute shrinkage and selection operator-Cox algorithm. The predictive accuracy of the risk model was evaluated through the Kaplan–Meier, receiver operating characteristic and Cox regression analyses. The performance of the model was verified in the International Cancer Genome Consortium-Liver Cancer - RIKEN, JP cohort. A nomogram was constructed to predict the 1-, 2-, 3-, and 5-year survival of HCC patients, and its performance was evaluated using calibration curves. The significantly enriched gene ontology terms, Kyoto encyclopedia of genes and genomes pathways and infiltrating immune cell populations associated with the IRG model were also analyzed to explore of the potential molecular mechanisms and immunotherapeutic targets. An independent and highly accurate prognostic model consisting of 7 IRGs was established and verified in 2 independent HCC cohorts. The IRG model was significantly associated with cell division and cell cycle. In addition, the high-risk group was more likely to have greater infiltration of immune cells and higher expression of immune checkpoint-related genes compared to the low-risk group. An IRG-based model was established to predict 1-, 2-, 3-, and 5-year survival rate in individual HCC patients, which provides new insights into the role of inflammasomes in HCC.

## 1. Introduction

Primary liver cancer is the 6^th^ most common malignancy, the third leading cause of cancer related mortality worldwide. Hepatocellular carcinoma (HCC) accounts for about 75% to 85% of primary liver cancer cases.^[1]^ HCC is routinely diagnosed on the basis of serum alpha-fetoprotein levels and the tumor node metastasis staging system, which are respectively limited by poor sensitivity and low accuracy.^[2,3]^ The conventional therapeutic modalities for HCC including surgical resection, liver transplantation, interventional therapy, or ablation.^[4]^ In recent years, targeted therapies and immune checkpoint inhibitor therapies have achieved favorable outcomes in HCC patients.^[5]^ Despite advances in the diagnosis and systemic treatment, HCC is highly heterogeneous at both clinical and molecular levels, and eventually still leading to high rates of recurrence and metastasis.^[6,7]^ Therefore, a reliable prognostic model is urgently needed to predict survival of HCC patients and guide clinical treatment.

Inflammasomes are multiprotein complexes consisting of a sensor protein, pro-caspase-1 and adaptor protein ASC that mediate innate immune responses. The inflammasomes are activated in response to pathogen-associated and damage-associated molecular patterns, which in turn triggers pro-caspase-1 activation and culminates in the release of the inflammatory cytokines interleukin (IL)-1β and IL-18, eventually triggering pyroptosis.^[8,9]^ Previous studies have shown that inflammasomes function as double-edged swords in cancer development.^[10]^ In some cancers, inflammasomes can facilitate the progression, occurrence and metastasis of the tumor cells.^[11]^ Sustained activation of inflammasomes and the overproduction of IL-1β in the tumor microenvironment attract MDSCs and other immunosuppressive cells. Luteoloside inhibited the proliferation, invasion and metastasis of HCC cells by suppressing the NLRP3 inflammasome.^[12,13]^ Conversely, moderate inflammation, and apoptosis and pyroptosis promoted by inflammasomes can slow cancer progression.^[14]^ For instance, IL-18 production activates the natural killer (NK) cells that exert cytotoxic effects on tumor cells. In addition, 17 β-estradiol (E2)-induced activation of the NLRP3 inflammasome and pyroptosis may repress HCC progression.^[12,15]^

As we all know, the initiation and promotion of HCC are highly associated with chronic inflammation, with approximately 80% of cases developing as a consequence of chronic liver disease progressing to fibrosis and finally hepatocarcinogenesis.^[16–18]^ Intense and chronic inflammation leads to liver cell death, dying hepatocytes release high amounts of damage-associated molecular patterns and therefore acceleration in hepatic inflammation and altering microenvironment.^[19]^ Therefore, analyzing the levels of inflammasome-related genes (IRGs) and their effect on the survival of HCC patients can provide new insights into HCC progression. There are few such studies. To this end, we constructed a novel IRG-related model to predict the prognosis of HCC patients and evaluate the role of the immune microenvironment.

## 2. Materials and methods

**Figure 1.**
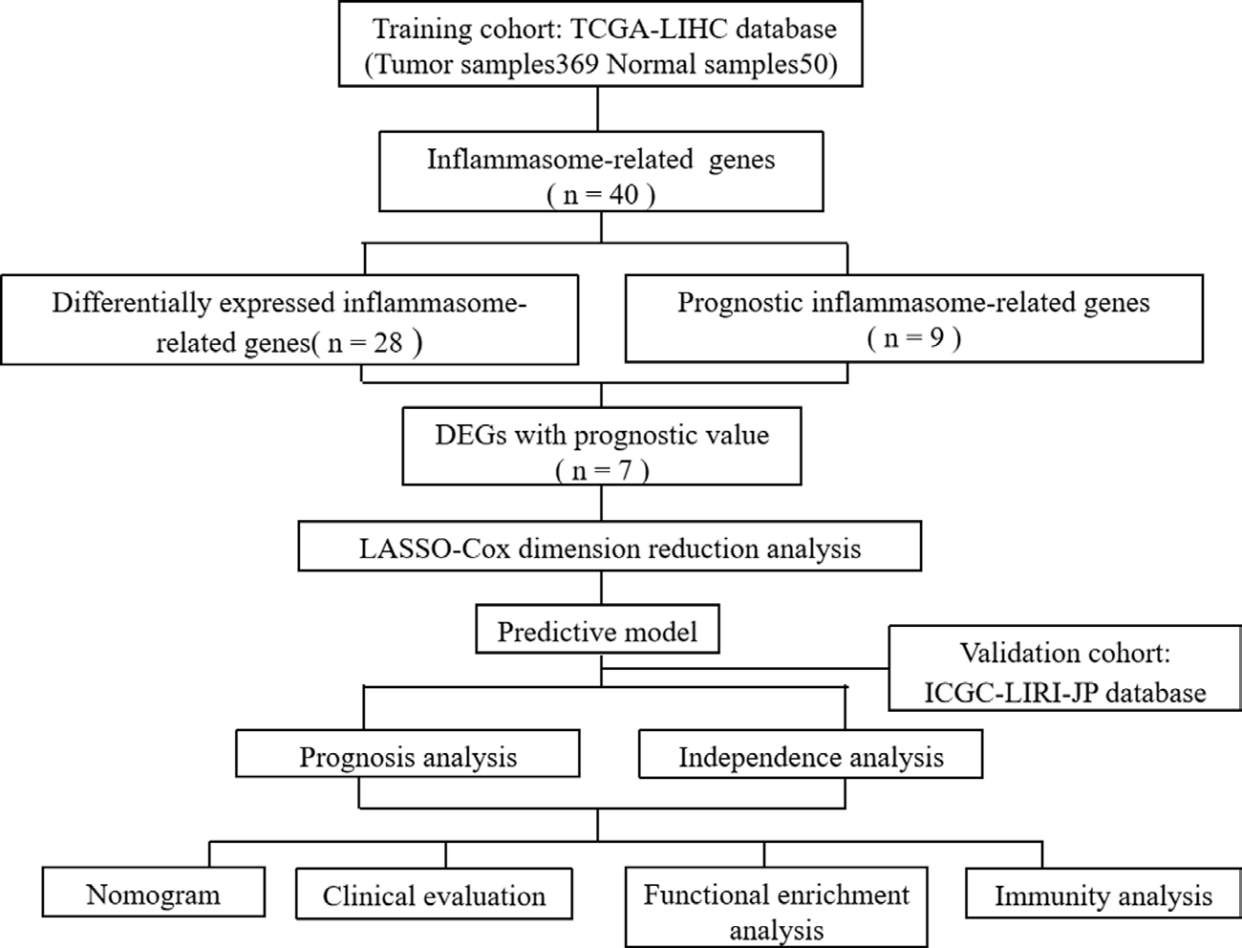
The flow chart of whole process of data analysis.

The overall workflow of this study is shown in Figure 1.

### 2.1. Data download and processing

The RNA-sequencing data and related clinical information of 374 HCC patients were downloaded from the cancer genome atlas (TCGA) website (https://xena.ucsc.edu/public/) as a training cohort. Likewise, the transcriptomics and clinical data of 232 HCC patients were downloaded from the International Cancer Genome Consortium-Liver Cancer - RIKEN, JP database as a validation cohort (https://dcc.icgc.org/projects/LIRI-JP). Patients lacking survival information were excluded.

### 2.2. Screening for prognosis-related DEIRGs

A total of 40 IRGs were retrieved from published literature (see Table S1, Supplemental Digital Content, http://links.lww.com/MD/I553, which illustrates inflammasome-related 40 genes).^[20]^

The differentially expressed genes between 369 HCC samples and 50 adjacent normal samples in TCGA cohort were screened using the “DESeq.2” R package. The intersecting genes between the differentially expressed genes and IRGs were identified as differentially expressed inflammasome-related genes (DEIRGs) (see Table S2, Supplemental Digital Content, http://links.lww.com/MD/I554, which illustrates 28 DEIRGs). The IRGs with prognostic value were further screened using univariate Cox analysis for overall survival (OS) (see Table S3, Supplemental Digital Content, http://links.lww.com/MD/I555, which illustrates 9 prognosis-related IRGs). The “pheatmap” and “forestplot” packages in R software were used to develop heatmaps and forest plots of the prognosis-related DEIRGs. In addition, the STRING database (https://string-db.org/) was used to generate a protein-protein interaction network of these DEIRGs.

### 2.3. Establishment and validation of IRG-based prognostic model

Least absolute shrinkage and selection operator-Cox dimension reduction analysis was performed using the “glmnet” R package. The λ value corresponding to the minimum partial likelihood deviance was selected, 7 candidate genes were obtained based on the OS in the training cohort. The lambda values of HSP90AB1, CASP7, CASP8, NLRC4, Mediterranean fever (MEFV), TXN, and TXNIP were 0.196, 0.193, 0.218, 0.491, 0.715, 0.112, and −0.197 respectively. The risk score of each patient was calculated as follows:

Risk score = expr_HSP90AB1_ × λ_HSP90AB1_ + expr_CASP7_ × λ_CASP7_ + expr_CASP8_ × λ_CASP8_ + expr_NLRC4_ × λ_NLRC4_ + expr_MEFV_ × λ_MEFV_ + expr_TXN_ × λ_TXN_ + expr_TXNIP_ × λ_TXNIP_, where expr_gene_ was the expression level of the gene and λ_gene_ the corresponding lambda value. Based on the median risk score, the patients were divided into the high-risk and low-risk groups. The “survival” package, “survminer” package and “time receiver operating characteristic (ROC)” package in R were used to perform Kaplan–Meier (KM) survival analysis and ROC analysis based on OS to estimate the prognostic precision of the model in both TCGA and ICGC cohorts. Finally, univariate and multivariate Cox regression analyses were performed to determine whether the risk score model can independently predict the prognosis of HCC patients.

### 2.4. Comparison of clinicopathological features between risk groups

The relationship between the risk score, clinicopathological features and the 7 candidate genes of the model was analyzed for each HCC patient in TCGA cohort using the “heatmap” R package. Wilcoxon signed-rank test was used to compare the risk score among patient subgroups demarcated on the basis of these clinicopathological features. The results were visualized in the form of boxplots.

### 2.5. Nomogram construction

A nomogram was constructed based on the risk score and clinical stage using the “rms” package in R to predict the 1-, 2-, 3-, and 5-year survival of HCC. Each factor was assigned a score, and the individual scores were summed up. The predictive accuracy was validated by plotting calibration curves.

### 2.6. Functional enrichment analysis

The most relevant genes of the risk score were uploaded to the Database for Annotation, Visualization, and Integrated Discovery, v6.8. The official gene symbol was selected as an identifier, and *Homo sapiens* was selected as the species. Gene ontology (GO) analysis and Kyoto encyclopedia of genes and genomes (KEGG) pathway analysis were performed. The top 5 items in ascending order of *P* value (*P* < .05) were displayed in the form of a bubble graph.

### 2.7. Immune infiltration analysis

Single-sample gene set enrichment analysis (ssGSEA) was performed to evaluate the degree of immune cell infiltration according to the expression levels of genes in 28 gene sets using the “gsva” module within R package.^[21]^ The expression of immune checkpoint-related genes in the 2 groups were analyzed using the “ggstatsplot” R package and visualized in the form of boxplots.

### 2.8. Human protein atlas

The immunohistochemistry images in the human protein atlas (HPA) database (https://www.proteinatlas.org/) were applied to validate the expression of prognostic DEIRGs in tumor and normal tissues.

### 2.9. Statistical analysis

Statistical analyses were performed using R (https://www.r-project.org/, V4.1.3). *P* < .05 was considered statistically significant.

## 3. Results

### 3.1. Identification of prognosis-related DEIRGs of TCGA cohort

**Figure 2.**
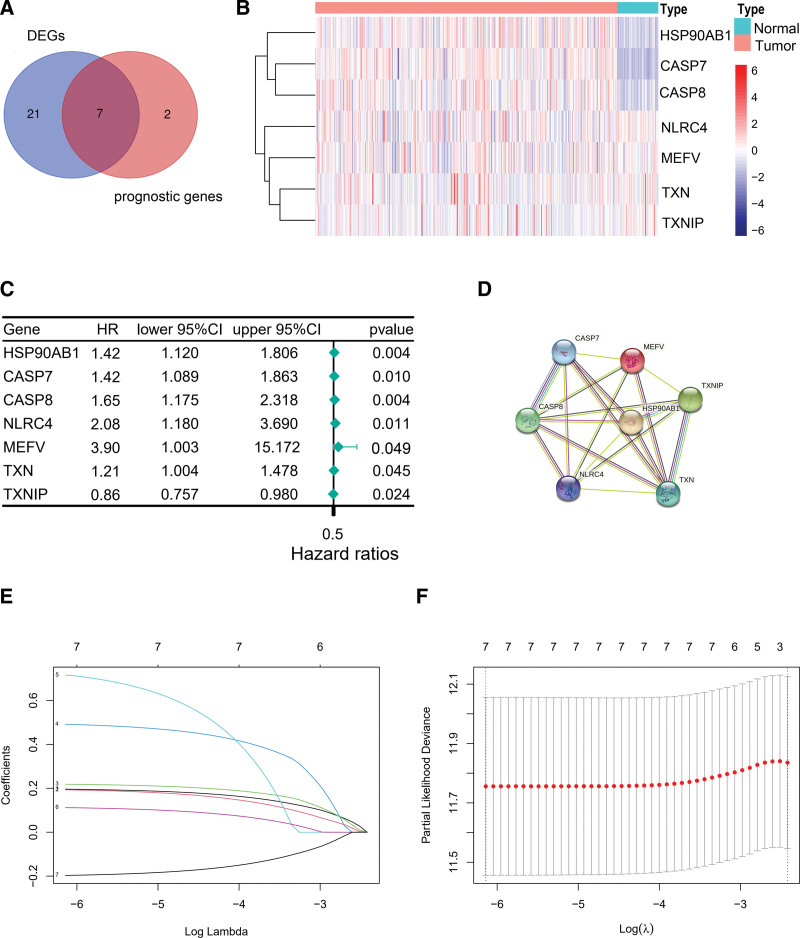
Identification of 7 optimal prognosis-related DEIRGs. (A) 7 identified prognostic DEIRGs. (B) The heatmap illustrated the differential expression of 7 prognosis-related DEIRGs between tumor samples and adjacent normal samples. (C) The Forest plot displayed the univariate Cox analysis results of prognosis-related DEIRGs. (D) The PPI network among 7 prognosis-related DEIRGs obtained from the STRING database. (E, F) Least absolute shrinkage and selection operator process of DEIRGs with survival prognostic value. DEIRGs = differentially expressed inflammasome-related genes, PPI = protein-protein interaction.

We retrieved 40 IRGs, of which 9 were related to prognosis. Among the latter, HSP90AB1, CASP7, CASP8, NLRC4, MEFV, TXN, and TXNIP were differentially expressed between the HCC and normal samples, and thus identified as prognosis-related DEIRGs (Fig. 2A). As shown in the heatmap in Figure 2B, HSP90AB1, CASP8 and TXN were upregulated, and CASP7, NLRC4, MEFV, and TXNIP were downregulated in the HCC tissues. The DEIRGs significantly associated with survival were screened through univariate Cox regression analysis (Fig. 2C). The protein-protein interaction network of these prognostic DEIRGs is shown in Figure 2D.

### 3.2. Construction of IRG-based prognostic model for HCC

A prognostic model based on the DEIRGs associated with survival was constructed by least absolute shrinkage and selection operator-Cox dimension reduction analysis (Fig. 2E and F). HSP90AB1, CASP7, CASP8, NLRC4, MEFV, TXN, and TXNIP were identified as candidate genes, and their respective lambda values were used to calculate the risk score for each patient.

**Figure 3.**
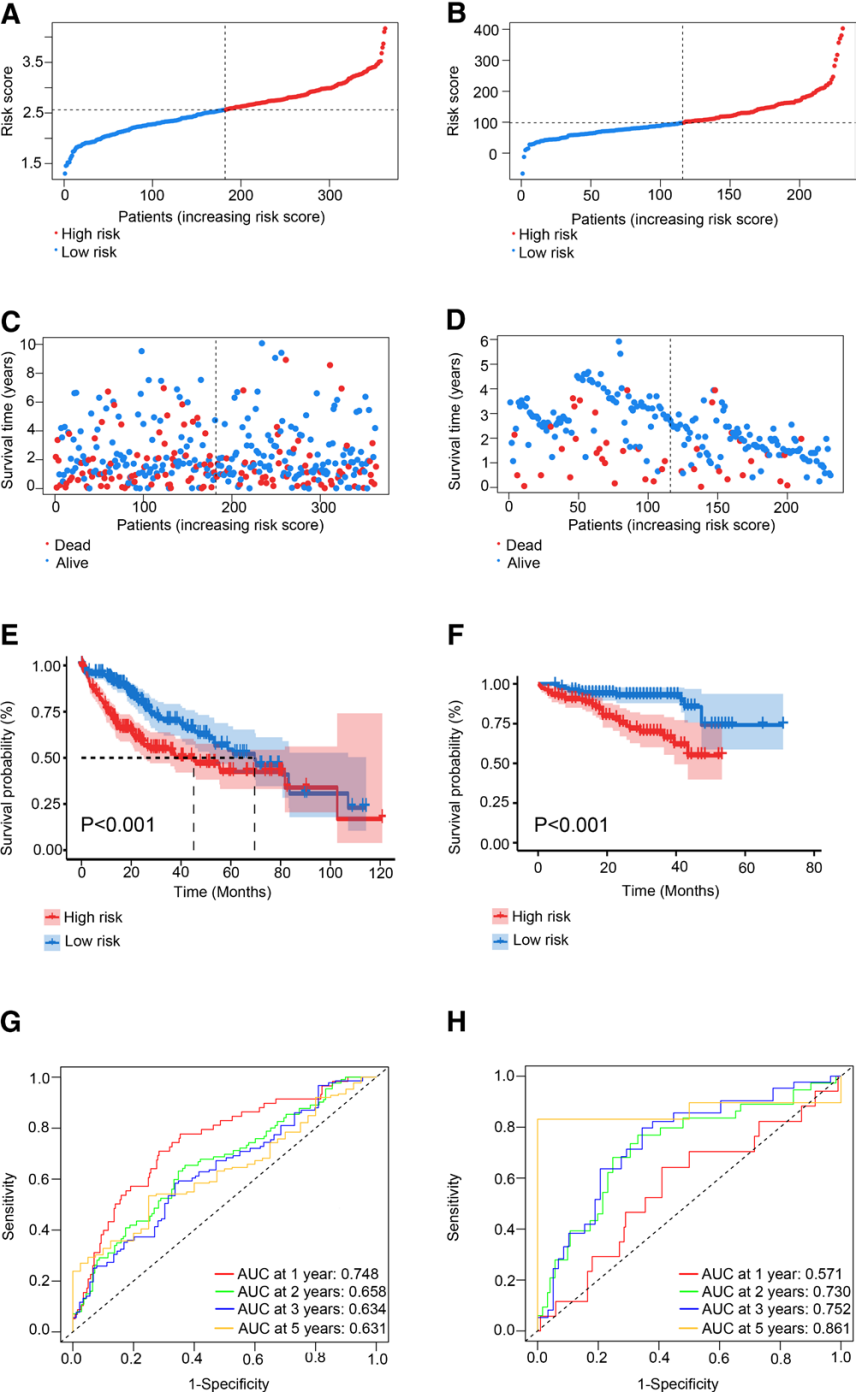
Construction and validation of the risk model in TCGA cohort and ICGC cohort. Distribution of patients based on the risk score in TCGA cohort (A) and ICGC cohort (B). Survival status scatter plots of patients in TCGA cohort (C) and ICGC cohort (D). The Kaplan–Meier curves displays the OS of patients between different risk groups in TCGA cohort (E) and ICGC cohort (F). Time-dependent ROC curves of the patients in TCGA cohort (G) and ICGC cohort (H). OS = overall survival.

Based on the median risk score, the patients in TCGA cohort were divided into the high-risk and low-risk groups. The high-risk score was associated with worse survival (Fig. 3A and C). In addition, KM analysis indicated that the patients with lower risk scores had higher survival rates (*P <* .001, Fig. 3E). The area under the time-dependent ROC curve (AUC) for 1-, 2-, 3-, and 5-year OS were 0.748, 0.658, 0.634, and 0.631 respectively (Fig. 3G), which suggests relatively high predictive accuracy of the risk score model. Furthermore, the model was validated in the ICGC cohort, and patients with higher risk score were more likely to have worse survival outcomes (*P <* .001, Fig. 3B, D, and F). As shown in the ROC curves in Figure 3H, the AUC for 1-, 2-, 3-, and 5-year OS in the validation cohort were 0.571, 0.730, 0.752, and 0.861 respectively. The above analysis proves that our model is stable across different platforms.

**Figure 4.**
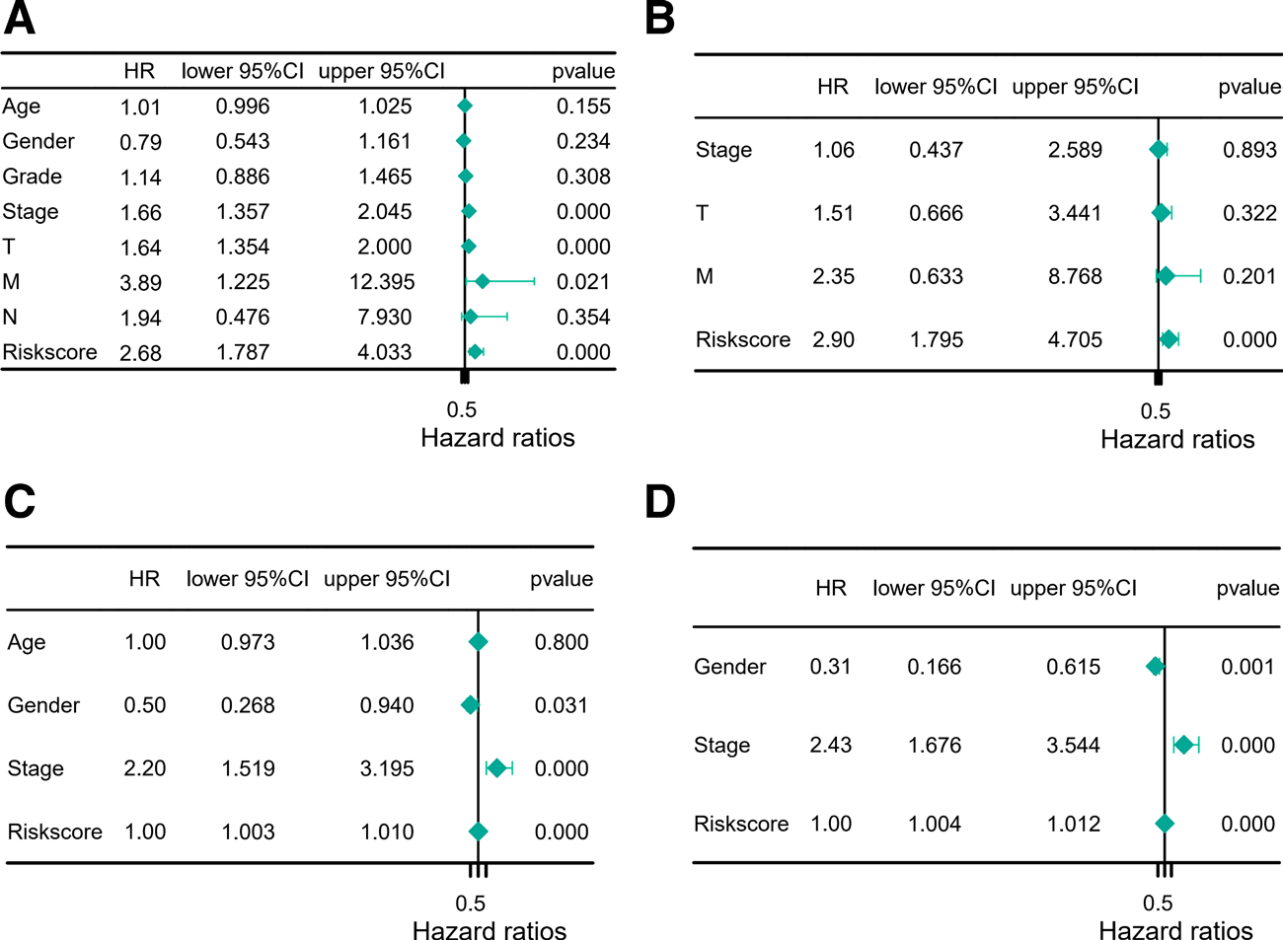
Independence confirmation of the model by DEIRGs. (A, B) The univariate and multivariate Cox regression results for TCGA cohort. (C, D) The univariate and multivariate Cox regression results for ICGC cohort. DEIRGs = differentially expressed inflammasome-related genes.

Univariate and multivariable Cox regression analyses were further performed to determine whether the DEIRG-based risk score is an independent prognostic factor in HCC (Fig. 4A and B). According to the univariate Cox analysis, the high-risk score was significantly associated with poor prognosis in HCC (*P <* .001, hazard ratio = 2.68, 95% confidence interval: 1.787–4.033), and identified as an independent risk factor by the multivariate Cox analysis (*P <* .001, hazard ratio = 2.90, 95% confidence interval: 1.759–4.705). The above results were also validated in the ICGC cohort (Fig. 4C and D).

### 3.3. Clinical evaluation of the IRG-based prognostic model

**Figure 5.**
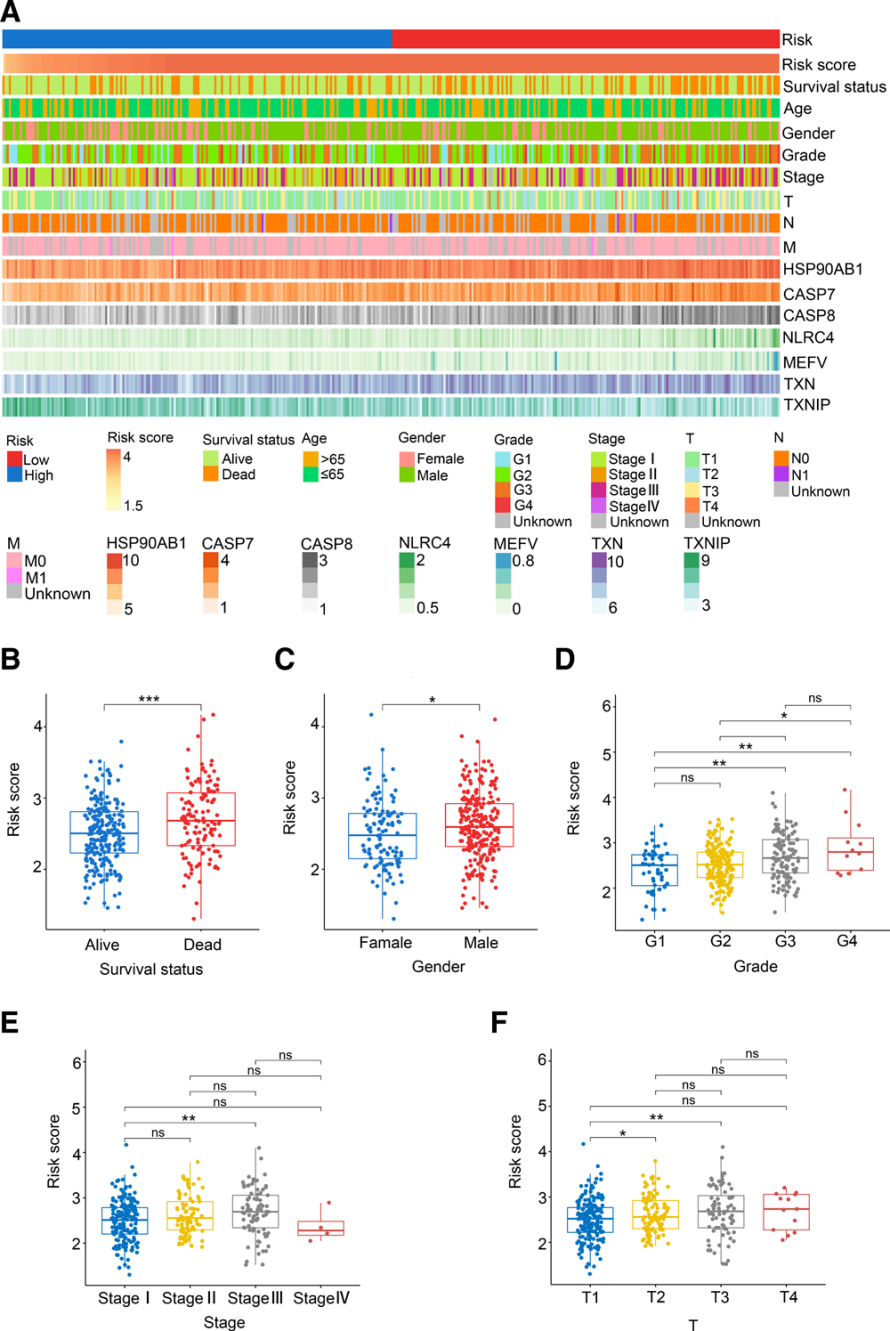
The relationship between risk score and clinicopathological features of HCC. (A) The heatmap showed the clinicopathological features and 7 representative genes for each HCC patient in ascending order of the risk score in TCGA cohort. (B–F) The boxplots demonstrated the differences in risk score across clinicopathological features.

The expression profiles of the 7 DEIRGs and clinicopathological features in TCGA cohort are shown in the heatmap in Figure 5A. The survival status, tumor grade and clinical stage showed asymmetric distribution with the increase in risk score. The patients were divided into subgroups on the basis of these clinicopathological characteristics, and the risk scores were compared by Wilcoxon signed-rank test. As shown in Figure 5B–F, the survival status, gender, clinical stage, tumor grade, and T stage were positively correlated to the risk score. The risk score was higher in the nonsurviving versus the surviving patients (*P <* .001). Furthermore, the male patients had a higher risk score compared to the female patients (*P <* .05). There was a significant upward trend of the risk score with the tumor grade increasing from G1 to G4 (*P* < .01), clinical stage from stage I to III (*P* < .01), the T stage from T1 to T3 (*P* < .01), and demonstrating that our risk model is strongly associated with these clinical factors.

### 3.4. Construction of a predictive nomogram for HCC

**Figure 6.**
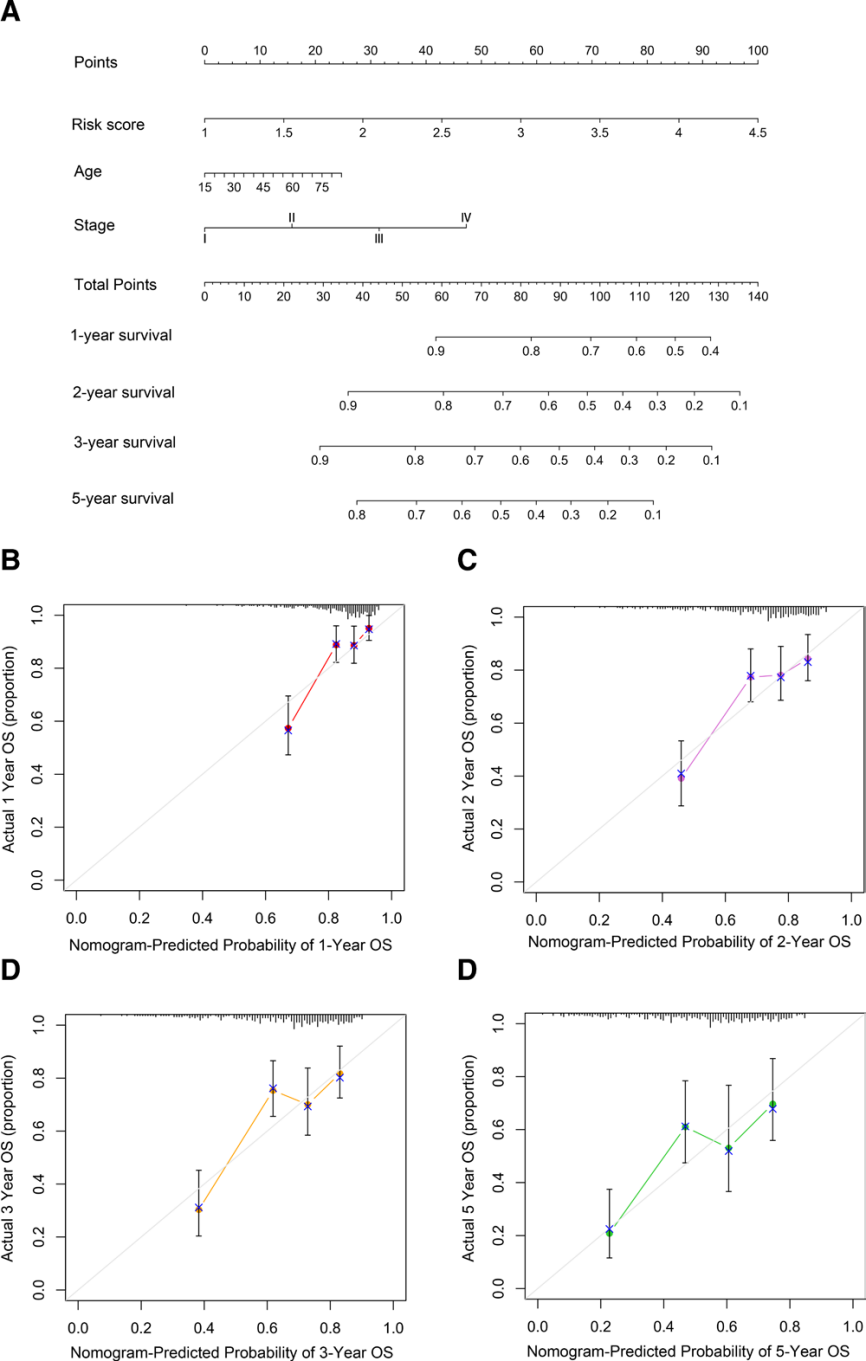
Prognostic nomogram was established by combining clinicopathological features and risk score. (A) Nomogram to predict the 1-year, 2-year, 3-year, and 5-year overall survival rate of HCC patients. (B–E) The calibration curves showed the comparison between predicted and actual overall survival for 1-, 2- 3-, and 5-year survival probabilities.

To facilitate the clinical application of the risk score model, we constructed a nomogram to predict the prognosis of individual patients. The nomogram for OS prediction was based on the independent predictive factors, including risk score, age, and clinical stage (Fig. 6A). The C-index of this nomogram model was 0.684. The calibration curve for predicting 1-, 2-, 3-, and 5-year survival showed that the nomogram and actual observations satisfactory overlap, indicating an optimal predictive accuracy (Fig. 6B–E).

### 3.5. Functional enrichment analysis of high-risk and low-risk groups

**Figure 7.**
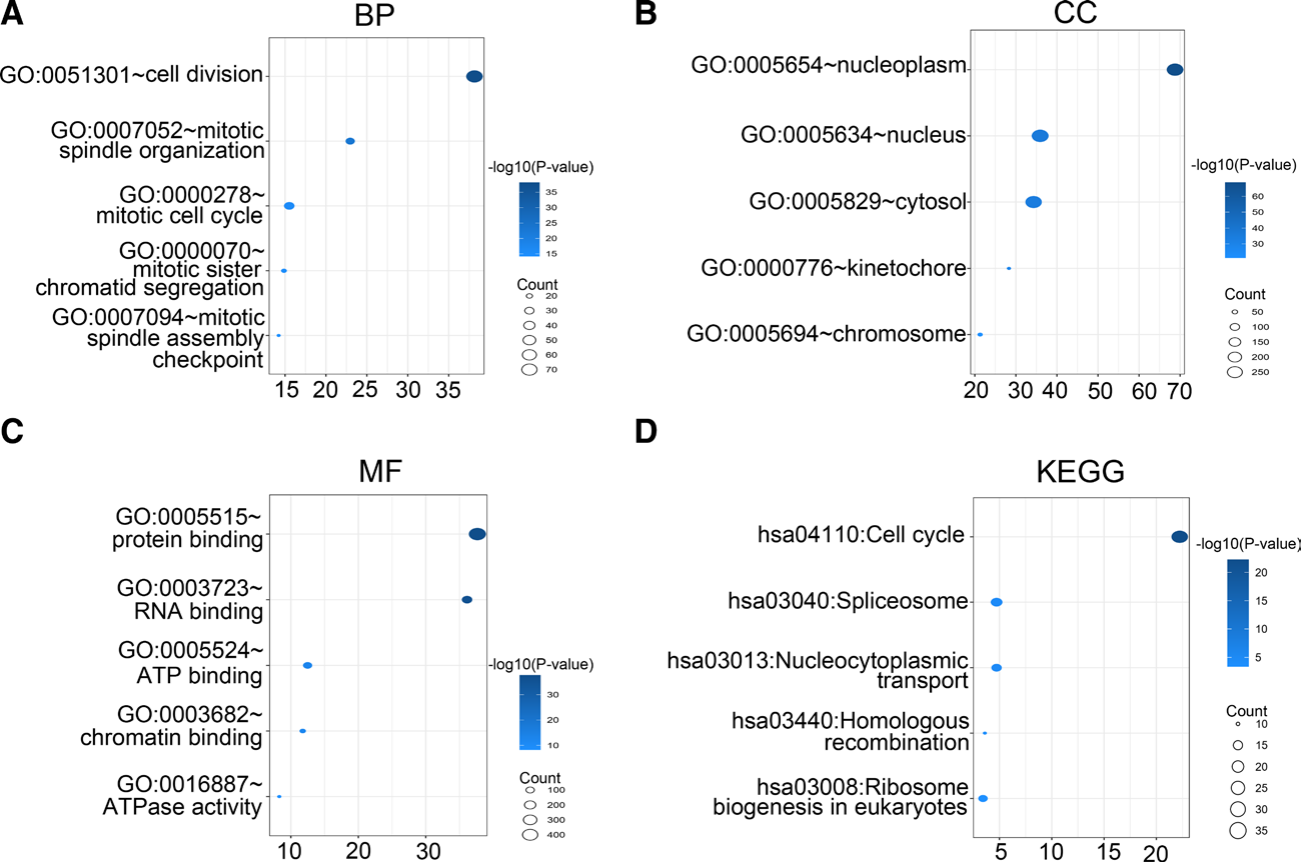
The functional analysis between the high- and low-risk groups in the TCGA. (A-C) Bubble graph for GO analysis, including biological process (BP), cellular component (CC), and molecular function (MF) of the model. (D) KEGG pathway enrichment of the model. GO = gene ontology, KEGG = Kyoto encyclopedia of genes and genomes.

To elucidate the biological functions and pathways associated with the risk score, we performed GO and KEGG pathway enrichment analyses on the DEIRGs between the high-risk and low-risk groups. The significantly enriched GO terms for biological processes were cell division, mitotic spindle organization and mitotic cell cycle, cellular component terms included nucleoplasm, nucleus and cytosol, and molecular functions associated with the DEIRGs were protein binding, RNA binding and ATP binding (Fig. 7A–C). Furthermore, KEGG analysis revealed that these DEIRGs mainly participate in cell cycle, spliceosome, nucleocytoplasmic transport, and other pathways (Fig. 7D).

### 3.6. The IRG-based risk model was significantly associated with the immune landscape of HCC

**Figure 8.**
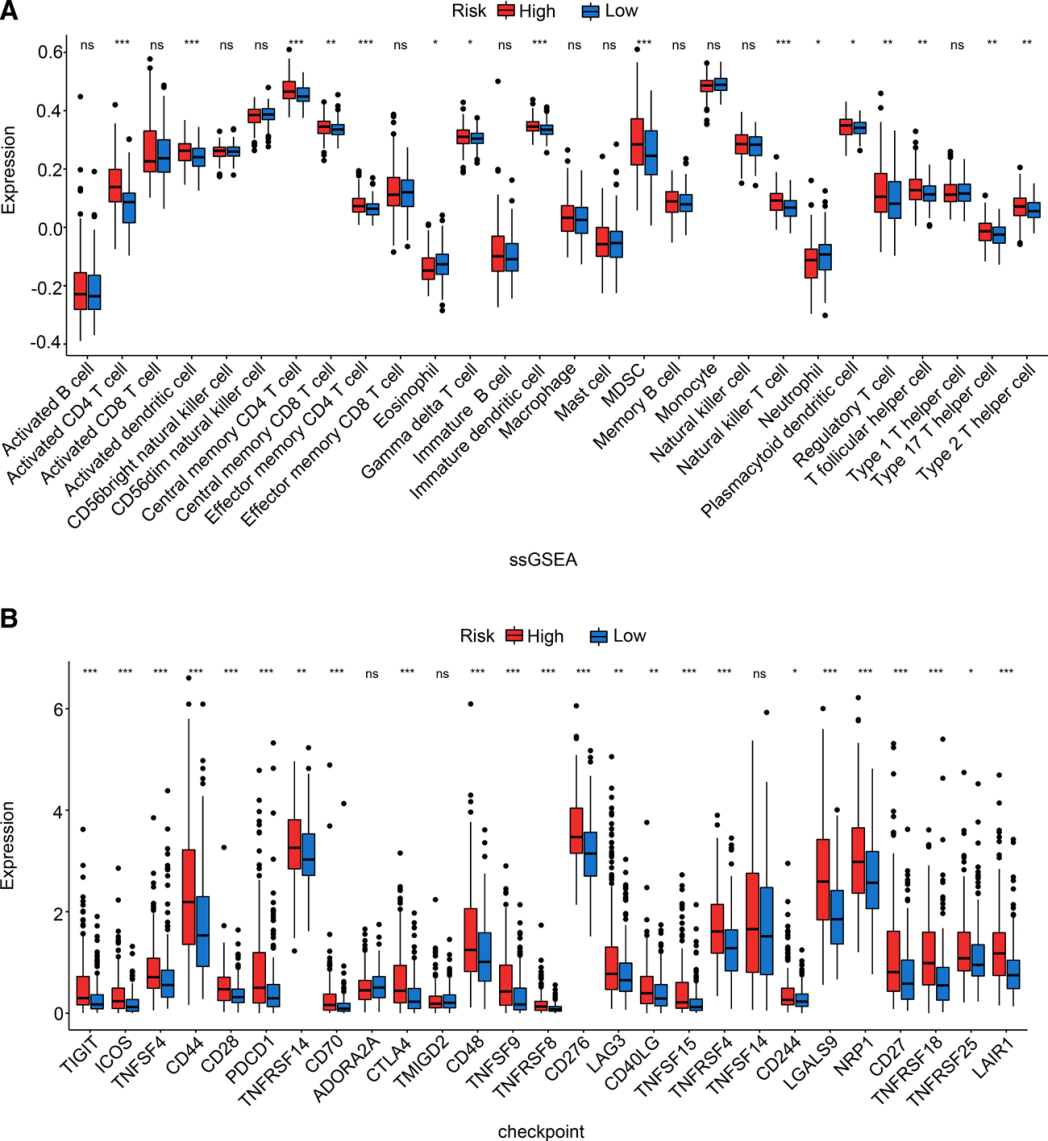
Immune characteristics analysis. (A) Expression of immune cells between different risk groups based on ssGSEA. (B) Expression difference of immune checkpoint-related genes between the 2 groups. ssGSEA = single-sample gene set enrichment analysis.

To further explore the immune landscape of both risk groups, the infiltration of different immune cell subpopulations was quantified through ssGSEA. As shown in Figure 8A, 14 immune cell subtypes (such as activated CD4 T cells, activated dendritic cells (DCs), MDSCs and regulatory T cells (Tregs)) were abundant in the high-risk group. Previous studies have shown that immune checkpoints play a crucial role in tumor immune escape. Therefore, we also examined the expression levels of immune checkpoint-related genes in the 2 risk groups. As shown in Figure 8B, the high-risk group was characterized by high expression of most immune checkpoint-related genes, including PDCD1, LAG3, CTLA-4, TIGIT, etc. Taken together, HCC patients with a high-risk score of IRGs may be more responsive to immunotherapies targeting the checkpoint molecules.

### 3.7. The protein expression levels of these prognostic DEIRGs in HCC tissues

**Figure 9.**
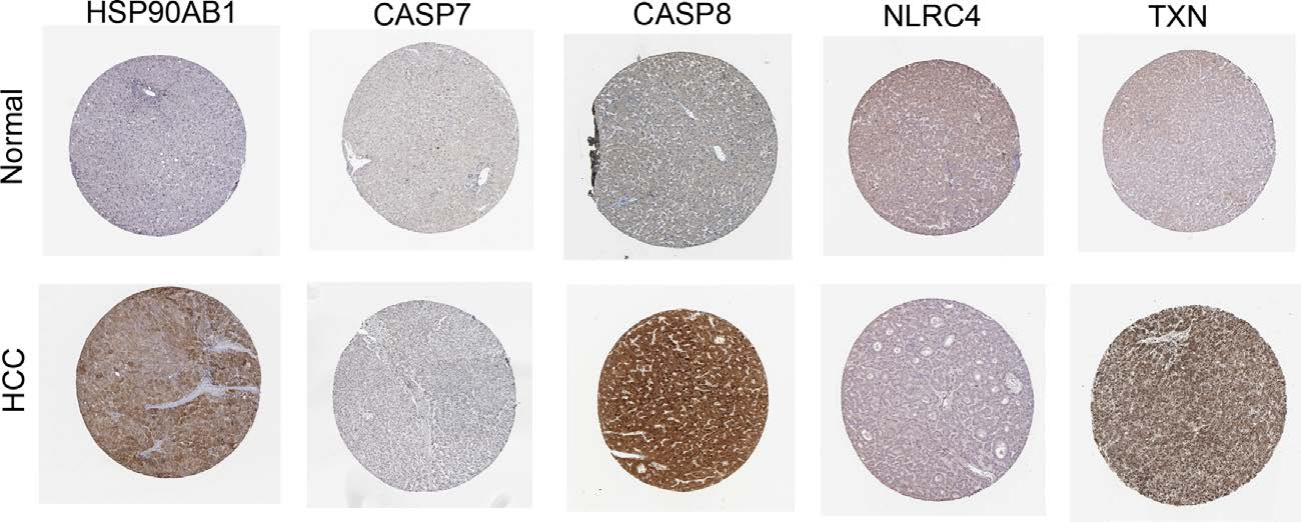
IHC of the HSP90AB1, CASP7, CASP8, NLRC4 and TXN in the normal and HCC tissues from the HPA database. HPA = human protein atlas, IHC = immunohistochemistry.

To validate the significant differences in protein expression levels of these prognostic DEIRGs between HCC tissues and normal tissues, we used an HPA database to assess the immunohistochemistry images. Consistently, the results demonstrated that the protein expression levels of HSP90AB1, CASP8, and TXN were higher in HCC tissues are comparable with normal tissues, while the expression level of CASP7 and NLRC4 were comparatively lower (Fig. 9). Unfortunately, the data of MEFV and TXNIP were not available in the HPA database.

## 4. Discussion

The complicated and vital roles of inflammasome-related genes, proteins and pathways in tumor genesis and development have garnered considerable attention.^[22]^ IL-1 signaling was identified to contribute to clonal expansion and progression of bone marrow fibrosis in myeloproliferative neoplasms disease.^[23]^ Inflammasomes, including NLRP3, caspase-1, NLRP1, NLRP6, NLRC4, and inhibit cancer development in colitis-associated colon cancer.^[24,25]^ Previous studies have also shown that the inflammasomes are involved in the progression from fibrosis to cirrhosis and ultimately liver cancer.^[18]^ Studies such as these have revealed that understanding the relationship between inflammasomes and tumor progression contributes to address many of the problems of tumor treatment and prognosis assessment. In this study, we performed bioinformatics analysis to develop a novel prognostic model for HCC based on IRGs expression.

We analyzed the expression levels and prognostic significance of 40 IRGs in HCC and found that 28 IRGs were differentially expressed in HCC tissues relative to the normal liver samples, of which 7 were associated with survival and used to construct the risk model. The patients were stratified into the high-risk and low-risk groups based on the median risk score. The survival outcomes were significantly different between the 2 risk groups, the time-dependent ROC analysis indicated that the risk score could accurately predict the survival of HCC patients in the training and validation cohorts. Furthermore, the risk score was also identified as an independent prognostic factor in HCC, and correlated positively with gender, tumor stage and tumor grade. Finally, the IRG risk score was incorporated into a nomogram along with age and tumor stage to identify patients at high-risk for HCC. Calibration curves showed that the predicted survival probability was consistent with the actual results.

The IRG model consisted of HSP90AB1, CASP7, CASP8, NLRC4, MEFV, TXN, and TXNIP. Previous studies have shown that HSP90AB1 is overexpressed in various tumors, promotes cancer development and progression by stabilizing the reprogramming of oncogenic proteins.^[26]^ Furthermore, HSP90AB1 can induce endothelial cell-dependent tumor angiogenesis by transcriptionally activating vascular endothelial growth factor receptors (VEGFRs) in HCC cells.^[27]^ CASP7 and CASP8 are key players in the apoptosis cascade. Sound et al found that inhibition of apoptosis due to inactivating CASP7 mutations is involved in the pathogenesis of various solid tumors in humans.^[28]^ CASP8 is also an established oncogene in some tumors,^[29]^ silencing CASP8 in murine models inhibited the progression of early HCC tumors.^[30]^ Thus, CASP7 and CASP8 are promising diagnostic markers for HCC. A retrospective study suggested that high expression of NLRC4 in the normal liver parenchyma surrounding tumor tissues was significantly correlated with worse prognosis of HCC patients.^[31]^ NLRC4-driven IL-1β secretion mediates production of vascular endothelial growth factor-A and tumor cell proliferation during fatty liver disease-induced metastasis.^[32]^ MEFV is a sensor protein that initiates assembly of the inflammasome complex. Studies related to Inflammatory bowel diseases indicated that MEFV, which was crucial for inflammasome activation and IL-18 release, thereby reducing inflammation and tumorigenesis.^[33]^ The pyrin protein encoded by the MEFV gene regulates caspase-1 activation and consequently IL-1β maturation in familial Mediterranean fever.^[34]^ TXN and TXNIP belong to the thioredoxin system, which maintains the redox state of cells. High TXN expression in multiple tumors is related to poor survival.^[35]^ Furthermore, Zhang et al^[36]^ revealed that silencing TXN triggered DNA damage response and cellular senescence under hypoxia in liver cancer cells. TXNIP, a negative regulator of thioredoxin, is downregulated in various aggressive cancers, and has been identified as a tumor suppressor gene.^[37–39]^ Furthermore, TXNIP overexpression in HCC cells induced apoptosis and inhibited proliferation.^[40]^ These genes have been identified as prognostic markers of HCC for the first time in this study.

GO and KEGG enrichment analyses revealed that the IRG model genes are mainly involved in cell division, nucleus, protein binding, and cell cycle signaling pathways. Cancer-associated mutations that impair cell cycle control allow uncontrolled and persistent cell division and proliferation. Thus, cell cycle kinases and checkpoints are suitable therapeutic targets for cancer.^[41,42]^ Besides, ssGSEA showed that the high-risk and low-risk groups differed significantly in terms of the infiltrating immune cell populations, especially CD4 + T cells, DCs, MDSCs, Tregs, Th2 cells, and Th17 cells. CD4 + T cells have been implicated in cancer immunity for their helper functions. The diverse functional polarization of them (e.g., Th1, Th2, Th17, and Tregs) was proven to affect tumor immunity obviously, playing a significant role in terms of clinical prognosis of patients.^[43,44]^ Previous reports indicate that DCs increased infiltration levels and are associated with relapse, compared with primary HCC.^[45]^ Furthermore, plasmacytoid dendritic cells exist in numerous primary as well as metastatic human neoplasms and maintain an immunosuppressive tumor microenvironment.^[46]^ MDSCs and Tregs are essential components of the immune suppressive tumor microenvironment. Both cell types protect tumor cells from immune surveillance of the host and favor tumor progression.^[47,48]^ The high-risk group was more likely to have relatively higher immune cell infiltration compared to the low-risk group, which is consistent with the function of inflammasomes. In addition, it is well-known that immune checkpoint blockers (ICBs) can improve the overall survival of a part of patients among different cancers.^[49]^ Moreover, Demaria O et al^[50]^ have observed that inflammation is a key factor in shaping ICB-triggered antitumoral immunity. And pharmacological activation of inflammasome components has been proposed as a potential strategy to augment the antitumor efficacy of ICBs.^[51,52]^ Notably, our data illustrated that immune checkpoints such as PDCD1, LAG3, CTLA-4, and TIGIT, etc were upregulated in the high-risk group, indicating that patients with high-risk score may be more responsive to immune checkpoint inhibitors.

The study has several limitations that ought to be discussed. For instance, the predictive value of the IRG model will have to be validated by including more individual clinical characteristics. Furthermore, inclusion of more clinical parameters and predictors can increase the prediction accuracy of the model. Since we did not validate the genes through in vitro and in vivo experiments, the pathways affected by these IRGs in HCC are still unclear and warrant further investigation.

In summary, we established a simple and accurate tool for predicting the survival probability of HCC patients, which can aid clinical decision making. Given that the model is closely related to the immune landscape of HCC, we will next explore the immunological pathways affected by the IRGs.

## Acknowledgments

We thank all participants for their time and effort.

## Author contributions

**Conceptualization:** Zhengqi Lv.

**Data curation:** Zhengqi Lv, Yiwen Yuan.

**Formal analysis:** Zhengqi Lv.

**Methodology:** Zhengqi Lv, Heng Li.

**Resources:** Zhengqi Lv.

**Software:** Zhengqi Lv.

**Validation:** Zhengqi Lv.

**Visualization:** Zhengqi Lv.

**Writing – original draft:** Zhengqi Lv.

**Writing – review & editing:** Zhengqi Lv, Qinghua Wu.

## Supplementary Material






