# Supplementary material for: A novel inflammasome-related gene nomogram predicts survival in hepatocellular carcinoma
Source: Medicine (Baltimore). 2023 Feb 22;102(8):e33121. doi: 10.1097/MD.0000000000033121 (PMC11309600; doi:10.1097/MD.0000000000033121)
Supplement: Supplementary file 1 [file medi-102-e33121-s001.pdf]

Supplemental Digital Content:

Table S1. A list of the inflammasome-related 40 genes in this study.

| | | | | |
|---|---|---|---|---|
| AIM2 | APP | BCL2 | BCL2L1 | CASP1 |
| CASP4 | CASP7 | CASP8 | CARD8 | HSP90AB1 |
| IFI16 | IL18 | IL1B | IL1RL1 | MEFV |
| NFKB1 | NFKB2 | NFKBIA | NLRC4 | NLRC5 |
| NLRP1 | NLRP2 | NLRP3 | NLRP6 | NLRP7 |
| NLRP12 | NAIP | PANX1 | PSTPIP1 | PYCARD |
| P2RX7 | RELA | SUGT1 | TLR1 | TLR7 |
| TNF | TNFAIP3 | TOLLIP | TXN | TXNIP |
