# Supplementary material for: A novel inflammasome-related gene nomogram predicts survival in hepatocellular carcinoma
Source: Medicine (Baltimore). 2023 Feb 22;102(8):e33121. doi: 10.1097/MD.0000000000033121 (PMC11309600; doi:10.1097/MD.0000000000033121)
Supplement: Supplementary file 2 [file medi-102-e33121-s002.pdf]

Supplemental Digital Content:

Table S2. 28 differentially expressed inflammasome-related genes in this study

APP	CASP1	CASP4	CASP7	CASP8
CARD8	HSP90AB1	IFI16	IL1B	IL1RL1
MEFV	NFKB1	NFKB2	NFKBIA	NLRC4
NLRC5	NLRP1	NLRP2	NLRP3	NLRP12
NAIP	PANX1	PYCARD	TLR1	TNF
TOLLIP	TXN	TXNIP		
