# Supplementary material for: A novel inflammasome-related gene nomogram predicts survival in hepatocellular carcinoma
Source: Medicine (Baltimore). 2023 Feb 22;102(8):e33121. doi: 10.1097/MD.0000000000033121 (PMC11309600; doi:10.1097/MD.0000000000033121)
Supplement: Supplementary file 3 [file medi-102-e33121-s003.pdf]

Supplemental Digital Content:

Table S3. 9 of the 40 inflammasome-related genes were related to prognosis.

Gene	HR	pvalue	95%CI L	95%CI H
**CASP7**	1.42	0.009	1.089	1.863
**CASP8**	1.65	0.003	1.175	2.318
**HSP90AB1**	1.42	0.003	1.119	1.806
**MEFV**	3.90	0.049	1.002	15.172
**NLRC4**	2.08	0.011	1.180	3.690
**NLRP6**	0.68	0.002	0.538	0.873
**RELA**	1.75	0.015	1.113	2.768
**TXN**	1.21	0.045	1.004	1.478
**TXNIP**	0.86	0.023	0.757	0.980
